# Factors influencing human papillomavirus school-based immunization in Alberta: A mixed-methods study protocol

**DOI:** 10.1371/journal.pone.0278472

**Published:** 2022-12-01

**Authors:** Jennifer Malkin, Lisa Allen Scott, Amanda Alberga Machado, Gary Teare, Joanne Snider, Syed Farhan Ali Tirmizi, Thilina Bandara, Mika Rathwell, Cordell Neudorf

**Affiliations:** 1 Public Health Evidence and Innovation Division, Provincial Population and Public Health, Alberta Health Services, Calgary, Alberta, Canada; 2 Department of Community Health Sciences, Cumming School of Medicine, University of Calgary, Calgary, Alberta, Canada; 3 Department of Oncology, Cumming School of Medicine, University of Calgary, Calgary, Alberta, Canada; 4 Communicable Disease Control Division, Provincial Population and Public Health, Alberta Health Services, Edmonton, Alberta, Canada; 5 Urban Public Health Network, Saskatoon, Saskatchewan, Canada; 6 School of Public Health, University of Saskatchewan, Saskatoon, Saskatchewan, Canada; 7 Community Health and Epidemiology, University of Saskatchewan, Saskatoon, Saskatchewan, Canada; Nazarbayev University School of Medicine, KAZAKHSTAN

## Abstract

More than 1,300 Canadians are diagnosed with cervical cancer annually, which is nearly preventable through human papillomavirus (HPV) immunization. Across Canada, coverage rates remain below the 90% target set out by the Action Plan for the Elimination of Cervical Cancer in Canada (2020–2030). To support this Plan, the Canadian Partnership Against Cancer has commissioned the Urban Public Health Network (UPHN) to coordinate a quality improvement project with Canada’s school-based HPV immunization programs. In Alberta, the UPHN partnered with Alberta Health Services (AHS) for this work. This study has one overarching research question: what are parent/guardian and program stakeholder perceived barriers, enablers and opportunities to immunization for youth as part of the school-based HPV immunization program in Alberta? This study uses a mixed-methods sequential explanatory design. A survey will be emailed to a sample of Albertans with children aged 11–17 years. Questions will be based on a Conceptual Framework of Access to Health Care. Subsequent qualitative work will explore the survey’s findings. Parents/guardians identifying as vaccine hesitant in the survey will be invited to participate in virtual, semi-structured, in-depth interviews. Stakeholders of the school-based immunization program will be purposively sampled from AHS’ five health zones for virtual focus groups. Quantitative data will be analyzed using SAS Studio 3.6 to carry out descriptive statistics and, using logistic regression, investigate if Framework constructs are associated with parents’/guardians’ decision to immunize their children. Qualitative data will be analyzed using NVivo 12 to conduct template thematic analysis guided by the Framework. Study results will provide insights for Alberta’s public health practitioners to make evidence-informed decisions when tailoring the school-based HPV immunization program to increase uptake in vaccine hesitant populations. Findings will contribute to the national study, which will culminate in recommendations to increase HPV immunization uptake nationally and progress towards the 90% coverage target.

## Introduction

Infection with human papillomavirus (HPV) is common, with upwards of 75% of Canadians having at least one infection in their lifetime [1]. HPV is a sexually-transmitted agent, and while most infections resolve on their own, there are certain high-risk HPV types that can cause cell abnormalities which can lead to cancer [2]. In Canada, HPV infection causes nearly all cases of cervical cancer, and is a cause of anogenital and oropharyngeal cancers [1, 2].

The Gardasil® 9 vaccine is extremely immunogenic and effective. More than 99% of vaccine recipients develop antibodies to all vaccine HPV types one month after completing the series [3]. The vaccine has also demonstrated greater than 96% efficacy at preventing cervical, vulvar and vaginal disease [3]. So far, research has shown that protection can last at least eight years [2].

**Fig 1:**
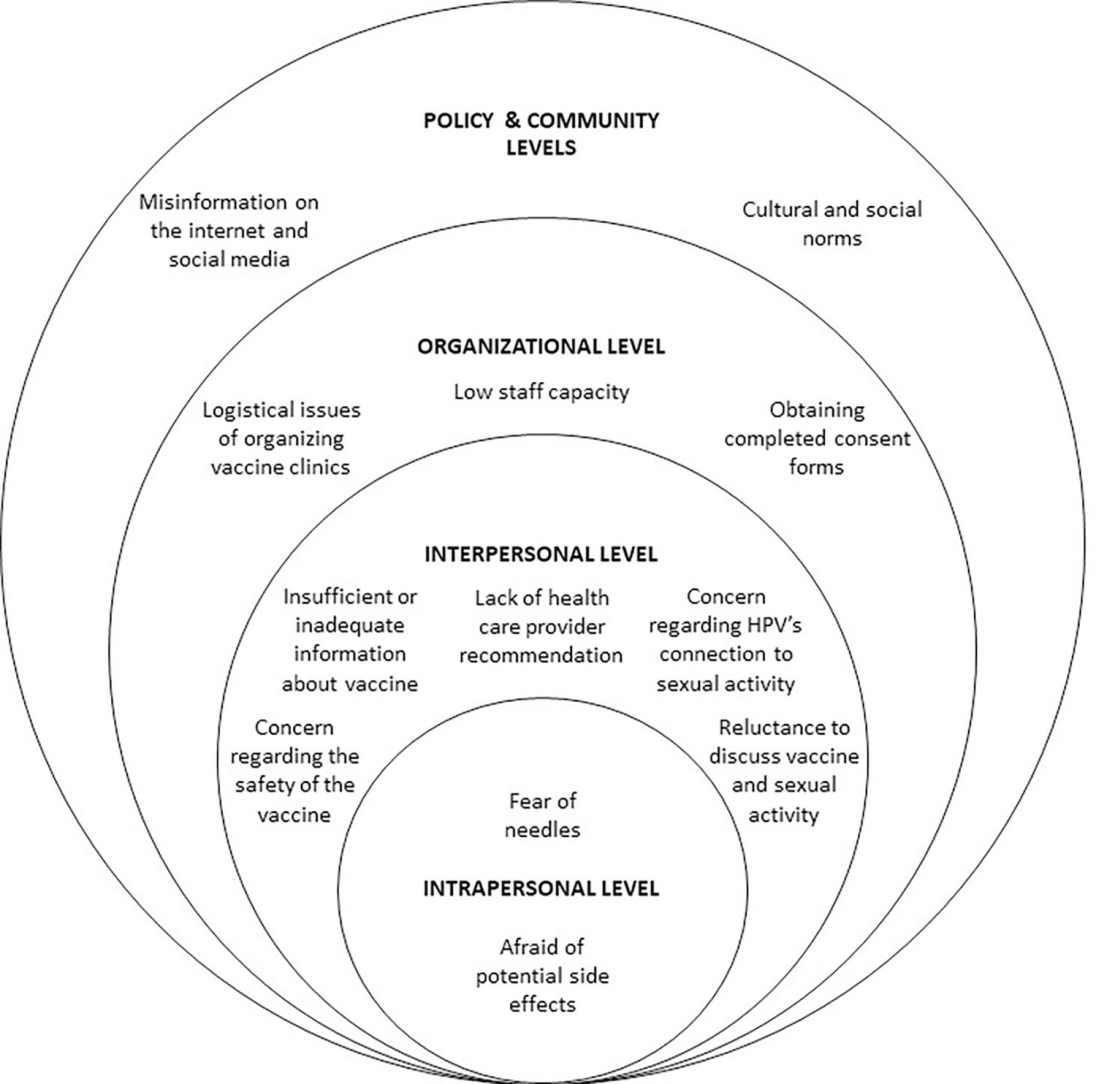
Barriers associated with HPV immunization based on the socio-ecological model [5].

If HPV immunization rates were to increase to 90%, we could expect to see cases of cervical cancer and related deaths to reduce by 23% and 21%, respectively [4]. If other HPV-related cancers that could be prevented through immunization were considered, there would be even greater benefits to both Canadians and the health care system. Yet, full immunization rates range from 57–91% in Canada’s provinces and territories (2015–2019) [1]. This has been reported to be associated with barriers at various socio-ecological levels (Fig 1) [5–30]. These low immunization rates have been exacerbated by the societal response to the COVID-19 pandemic [31, 32], highlighting the significance of examining and addressing these barriers.

Youth often are hesitant about receiving the HPV vaccine due to a fear of needles or possible side effects of the vaccine [6–8]. At the interpersonal level, prevalent barriers include lack of information, negative attitudes towards the vaccine, discomfort in talking about the vaccine, and absence of health care provider recommendation. Parents/guardians often have insufficient or inadequate information about the HPV vaccine [6, 9–13]; this issue can be exacerbated by language barriers, which may prevent full understanding of immunization program materials [9, 20, 22]. Some parents/guardians are apprehensive about the safety of the HPV vaccine [6, 10–17]. There is significant concern from parents/guardians regarding HPV’s connection to sexual activity. Parents/guardians are concerned that their child is too young to be receiving a vaccine for a sexually transmitted infection [6, 9, 11–13], that vaccine receipt will encourage their child to become sexually active [6, 10, 13], or cause complacency towards safe sex practices [6, 13]. Notably, recommendation from a health care provider also influences vaccine acceptance and uptake [11–13, 18–20]. Lastly, discussing the HPV vaccine can be a barrier to key groups: some parents/guardians are not comfortable with broaching the topic of sexuality with their child [6, 9]; teachers are often uncomfortable with promoting the HPV vaccine and answering student questions [9]; and health care professionals can be reluctant to discuss sexual activity with parents/guardians, especially those with strong religious beliefs [6].

At the organizational level, barriers involve vaccine staff capacity and immunization program logistics. Nurses feel as though they have little capacity to dedicate towards school immunizations due to their already high work load [7–9, 21]. A highly reported barrier is the issue of obtaining completed consent forms from parents/guardians [6, 8, 9, 15, 21, 22]. Other logistical issues relate to organizing immunization clinics within schools, including obtaining student immunization records, and running catch-up programs for students who miss immunization days [9, 21].

At the policy and community levels, barriers to immunization include misinformation, as well as cultural and social norms. Attitudes towards and understanding of the HPV vaccine have been adversely impacted by misinformation on the internet and social media [6, 9, 10, 17]. A systematic review on barriers to immunization among newcomers found that the HPV vaccine’s link to sexual health resulted in perceived conflict between immunization and one’s cultural beliefs [20]. In addition, social norms pertaining to gender roles, as well as the sourcing of health information, can impact the decision to immunize [20].

In 2020, the World Health Organization put forth a global call to action for the elimination of cervical cancer, and the Canadian Partnership Against Cancer (CPAC) has dedicated itself to doing so in Canada by 2040 [1]. Targeting barriers to HPV immunization will be critical in achieving this goal. Partners across Canada have collaborated to develop the Action Plan for the Elimination of Cervical Cancer in Canada (2020–2030), setting a target that by 2025, 90% of 17-year-olds will be fully immunized with the HPV vaccine. As part of this Action Plan, CPAC has commissioned the Urban Public Health Network (UPHN), along with the Public Health Physicians of Canada, to coordinate a quality improvement project with two objectives: (1) to describe the rates of HPV immunization across Canada; and (2) to understand the barriers and enablers that influence HPV immunization. The UPHN is a member-based non-profit organization of Medical Officers of Health representing 23 large cities across Canada, with a dedicated research group based out of the University of Saskatchewan. The UPHN is providing funds to local public health units across the country to catalyze HPV immunization program evaluation efforts. This protocol describes objective two, focusing on the process of learning about barriers and enablers to HPV immunization in Alberta, so that sustainable solutions, informed by priority populations and program stakeholders, can be identified and implemented.

### HPV immunization programs

Eligible Canadians can receive the HPV vaccine through two publicly-funded programs: the school-based and catch-up programs. Nationally, the HPV vaccine is offered to both girls and boys starting in grades four through seven, depending on the jurisdiction. Catch-up programs are available to those who did not receive the vaccine through a school-based program, or were in school before the introduction of the HPV vaccine, with eligibility varying by age and gender among the provinces and territories [1].

**Fig 2:**
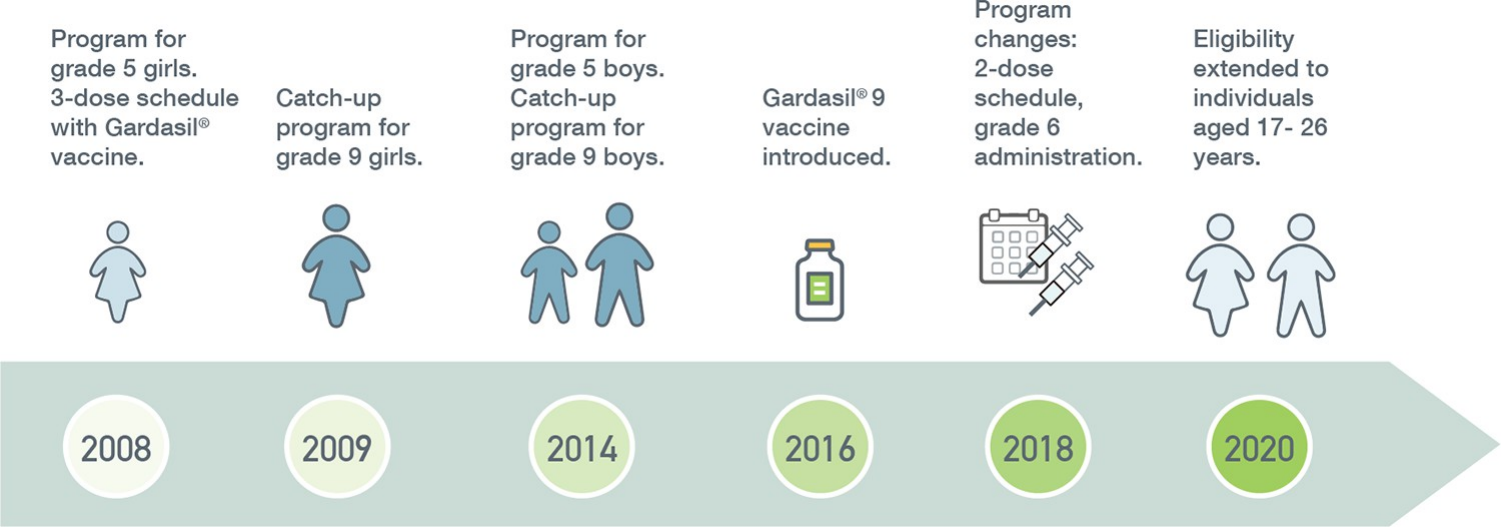
Evolution of Alberta’s HPV immunization program.

In Alberta, the HPV immunization program was introduced in 2008 (Fig 2). The Gardasil® vaccine was originally offered to females in grade five on a three-dose schedule over a six-month period. From school years 2009/10 to 2011/12, a catch-up program was introduced for females in grade nine. In 2014, vaccine eligibility was extended to include males in grade five, and a catch-up program was offered to males in grade nine from school years 2014/15 to 2017/18. The Gardasil® vaccine was replaced with the Gardasil® 9 vaccine in 2016. In 2018, the timing of vaccine administration was moved from grade five to six, and the number of doses was reduced from three to two for immunocompetent individuals aged nine to 14 years. In 2020, HPV vaccine eligibility was extended to include males and females aged 17 to 26 years, reducing the cost-associated barrier for this age group [33].

### Research questions

Despite widespread availability of the publicly-funded HPV vaccine, coverage in Alberta remains below the 90% target set out by CPAC [34]. To reconcile this coverage gap, barriers and enablers of HPV immunization must be identified and addressed. Public health professionals are aware that barriers and enablers may be idiosyncratic to different regions or sub-populations. To inform Alberta’s school-based HPV immunization program going forward, this study looks to understand how barriers and enablers vary across health zones and population groups.

This research study has one overarching research question: what are the perceived barriers and enablers to immunization as well as the opportunities to increase immunization for youth aged 11–15 years (grade six to nine) as part of the school-based HPV immunization program in Alberta through engagement with parents/guardians and program stakeholders? The quantitative component has the following research question: what are the perceptions and experiences of parents/guardians of school-aged youth with the school-based HPV immunization program? Then, the subsequent qualitative component has the following research question: how can the barriers, enablers and opportunities of the school-based HPV immunization program be described and explained from the perspective of parents/guardians and program stakeholders?

## Materials and methods

**Fig 3:**
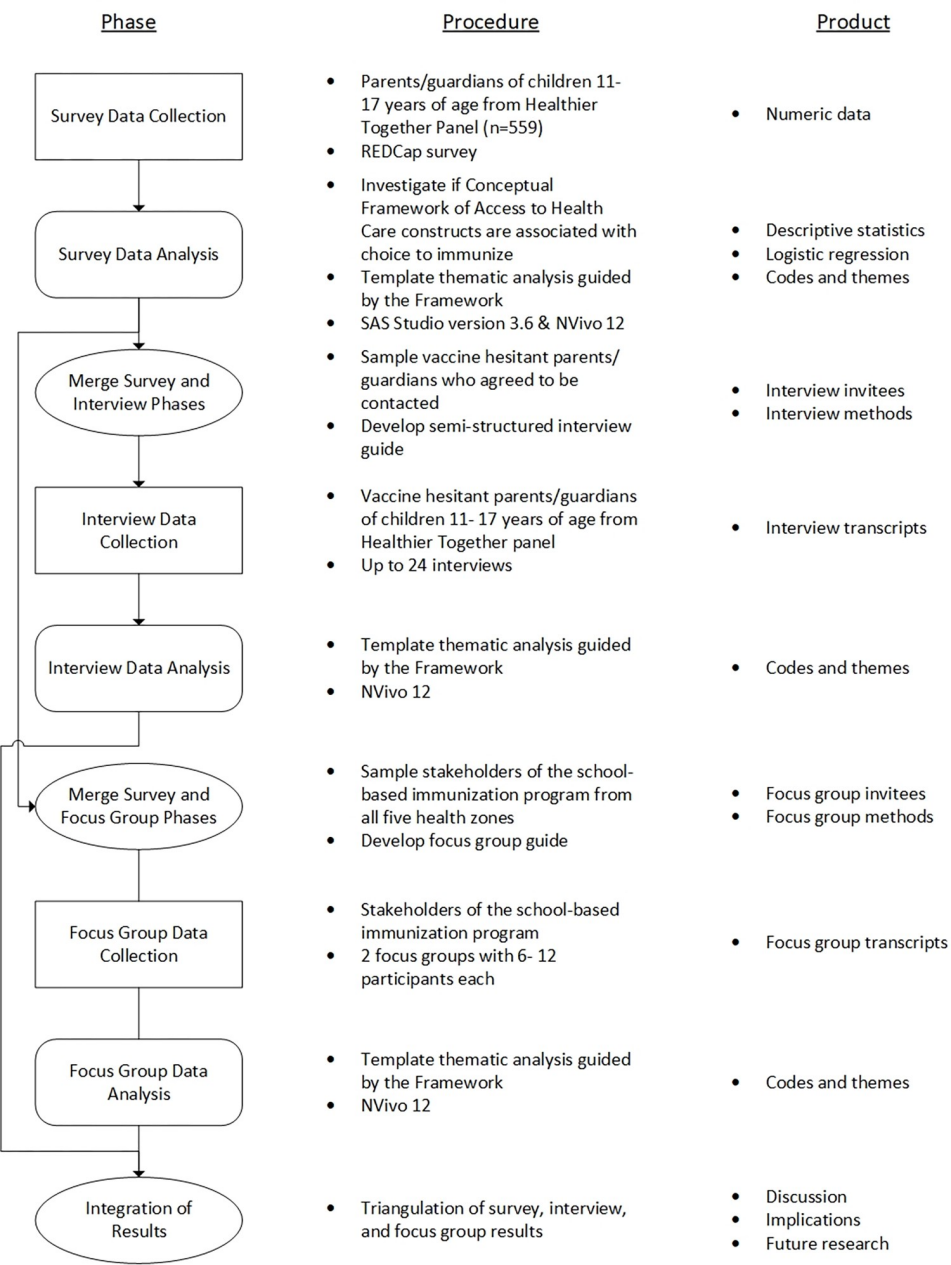
Visual of the mixed-methods sequential explanatory study design.

This study uses a mixed-methods sequential explanatory design (Fig 3) [35]. An initial survey will provide an overview of parent/guardian perceptions of HPV immunization and will be used to identify those who are vaccine hesitant. Parents/guardians that identify as vaccine hesitant in the survey will be invited to participate in a semi-structured, in-depth interview that seeks to further understand their perceptions of HPV and the vaccine. Furthermore, focus groups will be conducted with program stakeholders to understand barriers and enablers associated with the provincial program, health zone, and underserved sub-populations, which will be triangulated with those identified by parents/guardians. Through this approach, we will be able to understand parent/guardian and program stakeholder views more in-depth and provide insights for opportunities to adapt the school-based immunization program that will support increased HPV vaccine uptake.

### Conceptual framework of access to health care

**Fig 4:**
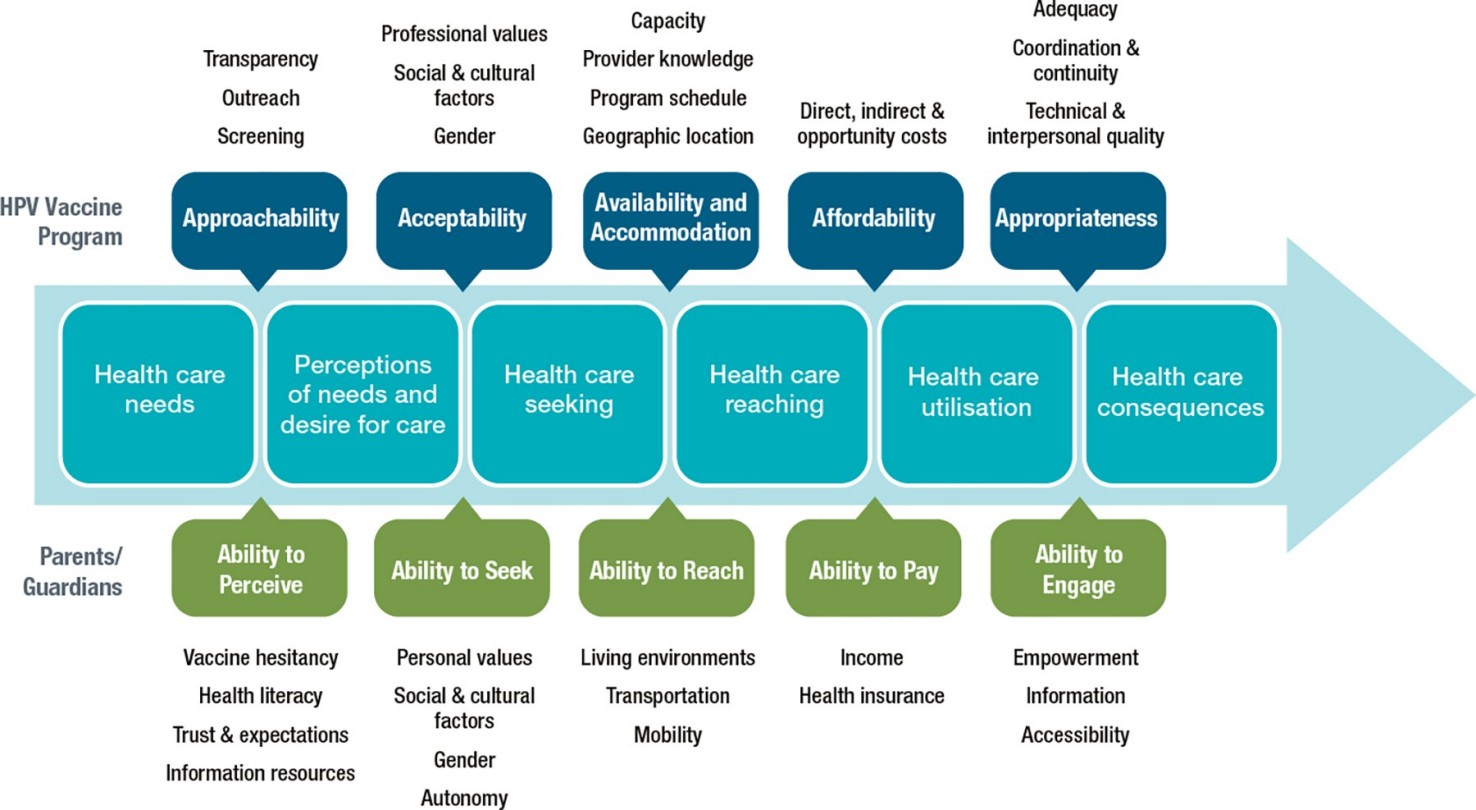
Conceptual framework of access to health care, adapted from Levesque, Harris & Russell [36].

The guiding framework for this study is the Conceptual Framework of Access to Health Care from Levesque, Harris and Russel (2013) (Fig 4) [36], which was developed based on a review of the literature on health care access. This Framework considers how the characteristics of both health services, and individuals, including the social determinants of health, interact to impact access to health care. This Framework describes five dimensions of health services, including: approachability, acceptability, availability and accommodation, affordability, and appropriateness. These dimensions interact with five abilities of individuals: ability to perceive; ability to seek; ability to reach; ability to pay; and ability to engage. This Framework will guide the development of data collection materials, as well as the quantitative and qualitative analyses.

### Setting

This study takes place in Alberta, Canada, which has a fully-integrated, publicly-funded health care system that delivers care to nearly 4.4 million people [37]. Alberta is separated into five health zones based on population, geography, and the distribution of health services: North, Edmonton, Central, Calgary and South. This study will investigate Alberta’s school-based HPV immunization program, which includes HPV vaccines administered by public health in schools across the province [38]. Currently, students are initially offered the HPV vaccine in grade six, and are screened for completion of the HPV vaccine series in grade nine.

### Population

There will be three populations of interest participating in this study. All participants must be residents of Alberta. The survey will involve a sample of parents/guardians of children 11 to 17 years of age recruited through the Healthier Together Panel of Albertans (description provided below). Semi-structured, in-depth interview participants will be those who answer in the survey that they chose not to immunize their child(ren) with the HPV vaccine and agree to be contacted about an interview. Focus group participants will be stakeholders of the school-based HPV immunization program who will be purposively sampled to achieve representation across all five health zones.

### Survey with parents/guardians

Survey participants will be recruited using the AHS Healthier Together Panel of Albertans. The panel consists of a diverse pool of approximately 5,000 individuals who have been pre-recruited across Alberta, and have agreed to participate in various data collection activities pertaining to population health. Using immunization data from the Interactive Health Data Application from Alberta Health [34], six Local Geographic Areas (LGAs) were identified for panel oversampling, four of which had low HPV vaccine coverage (<50%), while two had high coverage (>75%). LGAs with both low and high coverage were selected so as to increase the likelihood of learning about the barriers and enablers that influence immunization. LGAs were selected if they maintained consistent high or low coverage for both males and females from 2016 to 2018. Three LGAs were selected from both the North and South zones to increase the potential for comparisons. Panel oversampling was completed prior to study initiation in order to determine sample size, and as a part of routine panel maintenance and replenishment.

AHS Healthier Together panelists with children under the age of 18 will be sent an email inviting them to participate in a survey on the school-based HPV immunization program. The recruitment strategy will be limited by the fact that panelists are only asked whether they live with children under the age of 18, not how old these children are. To ensure that answers to survey questions are recently retrospective, the recruitment email will state that only panelists with children aged 11–17 years will be eligible to participate. Of the 5,000 panelists, 1,698 currently live with children under the age of 18; this will be the population that will be invited to participate in the survey. To determine sample size, we assumed that the proportion of parents/guardians who are vaccine hesitant is equivalent to the proportion of children who are not immunized for HPV in the school-based program. In 2018, the proportion of youth aged 12 who had not received two doses of the vaccine was 32% [34]. Based on the aforementioned information, and with a 95% confidence level and design effect of 2.0, a sample size of 559 panelists was calculated using the following equation: n = [design effect*Np(1-p)]/ [(d^2^/Z^2^_1-α/2_)*(N-1)+p*(1-p)] [39]. Although all 1,698 panelists will be invited to participate in the survey, the survey will close once the sample size has been achieved.

The survey was developed to address constructs of the Conceptual Framework of Access to Health Care. Questions inquire about the dimensions of health services and the abilities of individuals associated with the Framework, and were either written specifically for a component of Alberta’s school-based HPV immunization program, or were selected from a review of the literature. To establish face validity [40], the survey instrument was reviewed by staff in Communicable Disease Control (CDC), a division within AHS responsible for the provincial immunization program. The CDC team reviewed the survey for accuracy, relevancy and whether any major barriers or enablers were missed in the questions.

The survey is based on the Conceptual Framework of Access to Health Care [36], and consists of 60 questions, taking approximately 10–15 minutes to complete. The survey starts with asking for some preliminary information, including demographics (e.g., level of education completed, annual household income, ethnicity) and general HPV immunization questions, including whether they have immunized their child(ren) with the HPV vaccine. Answers to the latter question will determine sampling for the interviews and act as the dependent variable in analysis. For the *Ability to Perceive* construct, the following questions are included: the nine-item HPV-specific vaccine hesitancy scale, modified to be a five-point Likert scale [41]; questions about health literacy, including the five-item, four-point Likert scale Calgary Charter on Health Literacy scale, modified to be read from a parent’s/guardian’s perspective [42]; a question on how often particular resources are used to access health information [43]; a question regarding their trust in public institutions [34]; and questions about their experiences with health care and immunization [44]. Questions for the *Acceptability* and *Ability to Seek* constructs include: whether particular people/groups support HPV immunization [45–47]; and whether they have heard negative information about the HPV vaccine [45]. For the *Availability and Accommodation* and *Ability to Reach* constructs, questions ask about timing of immunization clinics, and the time allocated for review of vaccine materials (i.e., vaccine information sheet, consent form). While the HPV vaccine is free with the school-based program, a question asking whether any other costs associated with immunization prevented the parent/guardian from getting their child(ren) immunized is included for the *Affordability* and *Ability to Pay* constructs. For the *Ability to Engage* construct, questions include: self-assessed involvement in the decision to immunize [48]; if there is access to sufficient information to make an informed immunization decision [49], and whether the vaccine materials are easy to read and understand. Lastly, there is an open-ended question for parents/guardians to specify additional barriers to HPV immunization for their child(ren), and a question asking whether they are interested in being contacted for a follow-up interview.

Survey data will be collected and managed using REDCap electronic data capture tools hosted at Alberta Health Services [50, 51]. REDCap (Research Electronic Data Capture) is a secure, web-based software platform designed to support data capture for research studies. Emails will be sent to the AHS Healthier Together panelists with a link to a study information page, consent form and online survey. Panelists who consent to participate will be directed to the survey. The survey will be active for 45 days, or until the sample size has been reached, whichever comes first, with three reminder emails sent during the data collection period.

### Interviews with vaccine hesitant parents/guardians

Semi-structured, in-depth interviews with vaccine hesitant parents/guardians will be carried out after survey data analysis is complete. Up to 24 interviews will be completed, or until saturation is reached when analysis does not result in the emergence of new codes. Vaccine hesitant survey participants who agree to be contacted regarding an interview will receive an email inviting them to participate in an interview. Interviews will be scheduled with individuals who consent to participate.

An interview guide will be developed in collaboration with CDC staff. The guide will be based on data collection tools used in similar studies [52, 53], and revised based on survey results. The guide’s questions will broadly address the constructs of the Conceptual Framework of Access to Health Care [36], but inductive coding will enable us to identify the priority constructs.

Interviews will last approximately an hour in length and be held over Microsoft Teams. Interviews will be audio recorded and transcribed verbatim, with identifying information removed.

### Focus groups with stakeholders of the school-based immunization program

After survey data has been analyzed, focus groups will be conducted with stakeholders of the school-based HPV immunization program. Two focus groups with six to 12 participants each will be completed. Stakeholders will be purposively sampled to achieve representation across the five health zones. Separate focus groups will be held for leadership, such as program or area managers, and front-line workers, such as registered or public health nurses. Focus groups will be scheduled with consenting participants based on group availability.

A focus group guide will be developed in collaboration with CDC staff. The guide will be developed using data collection tools used by similar studies [52, 53], and revised based on survey results. A question will be included to inquire about whether there are specific sub-populations that are underserved by the school-based HPV immunization program, and how the program could be modified to better suit the needs of these populations. The guide’s questions will broadly address the constructs of the Conceptual Framework of Access to Health Care [36], but inductive coding will enable us to identify the priority constructs. The guide will be tailored depending on the role of the program stakeholder.

Focus groups will last approximately an hour to an hour and a half in length and take place over Microsoft Teams. Focus groups will be audio recorded and transcribed verbatim, with any identifying information removed.

### Analysis framework

The Conceptual Framework of Access to Health Care will be used to guide analyses of the three components of this mixed-method study [36]. By considering both parent/guardian and program stakeholder perspectives, we will be able to consider the five abilities of individuals, as well as the five dimensions of health services.

Quantitative survey data will be analyzed using SAS Studio version 3.6. We will carry out descriptive statistics and, using logistic regression, investigate if constructs of the Framework are associated with parents’/guardians’ decision to immunize their child(ren). Depending on answer distribution, Likert-scale questions may be dichotomized for analysis.

Qualitative data will be analyzed using NVivo 12. We will use template thematic analysis [54], identifying both descriptive and sentiment codes in the data, to ascertain barriers and enablers to HPV immunization and opportunities for how the school-based program could be improved. Where possible, results will be contextualized to AHS health zones or specific sub-populations. Codes will be inductively identified, with template themes framed around constructs of the Conceptual Framework of Access to Health Care. A trained Research Associate will code all transcripts.

### Integration of study results

Data from each study component will be triangulated to determine a holistic picture of the current realities of the school-based HPV immunization program and its opportunities for improvement. Findings on barriers and enablers in both quantitative and qualitative research components will be compared. Similarities and differences between the perspectives of parents/guardians and program stakeholders will also be assessed. If discrepancies in the findings are identified, they will be described, but with the more in-depth qualitative results taking precedence. We will map key themes to the Framework from both parent/guardian and provider perspectives to understand the pathway to increasing HPV immunization in Alberta. Attention will be paid to health equity and opportunities to tailor programming based on local contextual and population information.

Aggregated study results will be sent to the UPHN, where their research team will integrate these findings into their national efforts, which will culminate in recommendations to CPAC about where to best target resources to improve HPV immunization rates nationally.

### Ethics

This study was approved by the Health Research Ethics Board of Alberta (HREBA.CC-21-0364). The national quality improvement project conducted by the UPHN that will be using aggregate findings for the purposes of programmatic recommendations has received ethical exemption from the University of Saskatchewan’s Biomedical Research Ethics Board. To protect the confidentiality of future participants, data will not be shared publicly post study completion.

Signed consent will be obtained from all individuals prior to them participating in the study. Emails will be sent to recruited individuals with a document describing the objectives of the study and what their participation would look like. If they are interested in participating, they will be invited to complete a virtual consent form on REDCap.

Initial results will be shared back with respective consenting participants of the surveys, interviews and focus groups to confirm that their perspectives have been accurately described. Final study results will be shared with all consenting participants.

## Discussion

### Significance of the proposed study

Improving HPV immunization acceptance and uptake can significantly reduce the incidence of various cancers, particularly cervical cancer. Underserved populations are disproportionately impacted by cervical cancer, including Indigenous Peoples, newcomers, women with low income, and those living in rural and remote areas [1]. Newcomers, for example, may face barriers at various socio-ecological levels to vaccine acceptance and uptake, which may explain this disparity. At the interpersonal level, newcomers may lack sufficient vaccine information, particularly due to language barriers, and at the policy and community levels, there may be conflicts with cultural or social norms [20]. This is a significant sub-population to take into consideration when public health planning in Alberta, with immigrants and recent immigrants representing 21% and 5% of the population in 2016, respectively [55]. By identifying and addressing barriers to immunization in Alberta, we can hope to address these inequities when intervention planning.

As illustrated by the Conceptual Framework of Access to Health Care (Fig 4), access is multi-faceted, impacted across the levels of the socio-ecological model. By triangulating parent/guardian and program stakeholder perspectives, we hope to gain a comprehensive understanding of the challenges and opportunities of Alberta’s school-based immunization program. In Alberta’s school-based program, individuals under the age of 18 are considered minors without capacity [56], and therefore consent from a parent/guardian must be obtained prior to administering the HPV vaccine in the school setting. Thus, it is critical to consider and address the barriers that may be holding parents/guardians back from providing consent for their child(ren) to receive the HPV vaccine. In addition, recommendation of the vaccine from a health care provider is a significant factor in parents’/guardians’ decision to immunize their child(ren) [11–13, 18, 19], so it is important to gain a better understanding of the attitudes and perspectives of program providers, such as public health nurses, who discuss HPV immunization with parents/guardians.

Study findings will inform recommendations for both provincial and national partners. Triangulated study results will provide insights for Alberta’s public health practitioners to make evidence-informed decisions when tailoring the school-based HPV immunization program to increase uptake in vaccine hesitant populations. Study findings will inform CPAC of the current realities and priorities as they seek to create new policies, programs and funding opportunities.

### Strengths and limitations

This proposed study will provide insights into Alberta’s school-based HPV immunization program. By utilizing a mixed-methods sequential explanatory design, we will be able to both quantify and elaborate on the barriers and opportunities of the program. In addition, through considering both parent/guardian and program stakeholder perspectives, we will be able to consider all constructs of the Conceptual Framework of Access to Health Care. An additional strength of this study is its involvement in a national research program. Findings from other provinces/territories may bolster our own findings, supporting our understanding of how demographic and geographic characteristics impact acceptance and uptake of HPV immunization.

Despite these strengths, the proposed study also has some limitations. One limitation may be that the AHS Healthier Together panel may not be representative of the diversity within vaccine hesitant parents/guardians, which could impact generalizability of findings. To address this potential limitation, four LGAs that have demonstrated consistent low HPV coverage in both males and females over time were selected for panel oversampling. Since the survey will be self-administered and inquires about immunization, which can be a controversial topic for some folks, social desirability bias is another potential limitation of this study. Fortunately, we should be able to overcome this response bias with our interviews as this will provide us with the opportunity to probe participants to further understand inherent biases. A third limitation is that the survey instrument was not assessed for content validity or reliability [40]; however, the survey will be an exploratory tool to gain a baseline understanding of perspectives and experiences so that they can be further investigated in the subsequent qualitative work. If valuable, the survey instrument could undergo additional tests of validity and reliability for wider applicability. Finally, this study may also be limited by the fact that it is only targeted at Alberta’s school-based immunization program and its immediate stakeholders. Other settings and groups, such as primary care and GPs, significantly influence HPV immunization and it is important to take them into consideration; however, this was outside the scope of the current study, but could be the focus of future work. In addition, as this study only focused on the school-based program in Alberta, the results of this study cannot be extrapolated to other contexts. The results from the Alberta study, however, will be compared with the results from other participating provinces/territories to inform the national perspective, offering greater generalizability.

This study is currently undergoing participant recruitment and data collection and should be concluding by fall 2022.

## References

[1] Canadian Partnership Against Cancer. *HPV immunization for the prevention of cervical cancer*, https://s22457.pcdn.co/wp-content/uploads/2021/04/HPV-immunization-prevention-cervical-cancer-EN.pdf (2021).

[2] Canadian Cancer Society. What do I need to know about HPV?, https://cancer.ca/en/cancer-information/reduce-your-risk/get-vaccinated/human-papillomavirus-hpv.

[3] Chatterjee A. The next generation of HPV vaccines: nonavalent vaccine V503 on the horizon. *Expert Rev Vaccines* 2014; 13: 1279–1290. doi: 10.1586/14760584.2014.963561 25256262

[4] Canadian Partnership Against Cancer. *Cancer System Performance: 2018 Report*. Toronto (ON), https://s22457.pcdn.co/wp-content/uploads/2019/01/2018-Cancer-System-Performance-Report-EN.pdf (2018).

[5] McLeroy KR, Bibeau D, Steckler A, et al. An ecological perspective on health promotion programs. *Health Educ Q* 1988; 15: 351–377. doi: 10.1177/109019818801500401 3068205

[6] Ferrer HB, Trotter C, Hickman M, et al. Barriers and facilitators to HPV vaccination of young women in high-income countries: a qualitative systematic review and evidence synthesis. *BMC Public Health* 2014; 14: 700. doi: 10.1186/1471-2458-14-700 25004868 PMC4100058

[7] Grandahl M, Tydén T, Rosenblad A, et al. School nurses’ attitudes and experiences regarding the human papillomavirus vaccination programme in Sweden: a population-based survey. *BMC Public Health* 2014; 14: 540. doi: 10.1186/1471-2458-14-540 24886332 PMC4061918

[8] Gottvall M, Tydén T, Larsson M, et al. Challenges and opportunities of a new HPV immunization program perceptions among Swedish school nurses. *Vaccine* 2011; 29: 4576–4583. doi: 10.1016/j.vaccine.2011.04.054 21549793

[9] Dubé E, Gagnon D, Clément P, et al. Challenges and opportunities of school-based HPV vaccination in Canada. *Hum Vaccin Immunother* 2019; 15: 1650–1655. doi: 10.1080/21645515.2018.1564440 30633622 PMC6746476

[10] Scott K, Lou Batty M. HPV Vaccine Uptake Among Canadian Youth and The Role of the Nurse Practitioner. *J Community Health* 2016; 41: 197–205. doi: 10.1007/s10900-015-0069-2 26245727

[11] Ogilvie G, Anderson M, Marra F, et al. A population-based evaluation of a publicly funded, school-based HPV vaccine program in British Columbia, Canada: parental factors associated with HPV vaccine receipt. *PLoS Med* 2010; 7: e1000270. doi: 10.1371/journal.pmed.1000270 20454567 PMC2864299

[12] Rodriguez SA, Mullen PD, Lopez DM, et al. Factors associated with adolescent HPV vaccination in the U.S.: A systematic review of reviews and multilevel framework to inform intervention development. *Prev Med (Baltim)* 2020; 131: 105968. doi: 10.1016/j.ypmed.2019.105968 31881235 PMC7064154

[13] Loke AY, Kwan ML, Wong Y-T, et al. The Uptake of Human Papillomavirus Vaccination and Its Associated Factors Among Adolescents: A Systematic Review. *J Prim Care Community Health* 2017; 8: 349–362. doi: 10.1177/2150131917742299 29161946 PMC5932744

[14] Highet M, Jessiman-Perreault G, Hilton E, et al. Understanding the decision to immunize: insights into the information needs and priorities of people who have utilized an online human papillomavirus (HPV) vaccine decision aid tool. *Can J Public Heal* 2021; 112: 191–198. doi: 10.17269/s41997-020-00425-z 33078333 PMC7571294

[15] Dubé E, Wilson S, Gagnon D, et al. ‘It takes time to build trust’: a survey Ontario’s school-based HPV immunization program ten years post-implementation. *Hum Vaccin Immunother* 2021; 17: 451–456. doi: 10.1080/21645515.2020.1775456 32643527 PMC7899628

[16] Gilbert NL, Gilmour H, Dubé È, et al. Estimates and determinants of HPV non-vaccination and vaccine refusal in girls 12 to 14 y of age in Canada: Results from the Childhood National Immunization Coverage Survey, 2013. *Hum Vaccin Immunother* 2016; 12: 1484–1490. doi: 10.1080/21645515.2016.1153207 26942572 PMC4964714

[17] Kashani BM, Tibbits M, Potter RC, et al. Human Papillomavirus Vaccination Trends, Barriers, and Promotion Methods Among American Indian/Alaska Native and Non-Hispanic White Adolescents in Michigan 2006–2015. *J Community Health* 2019; 44: 436–443. doi: 10.1007/s10900-018-00615-4 30661151 PMC7457164

[18] Cheruvu VK, Bhatta MP, Drinkard LN. Factors associated with parental reasons for ‘no-intent’ to vaccinate female adolescents with human papillomavirus vaccine: National Immunization Survey—Teen 2008–2012. *BMC Pediatr* 2017; 17: 52. doi: 10.1186/s12887-017-0804-1 28193249 PMC5307730

[19] Krawczyk A, Knäuper B, Gilca V, et al. Parents’ decision-making about the human papillomavirus vaccine for their daughters: I. Quantitative results. *Hum Vaccin Immunother* 2015; 11: 322–329. doi: 10.1080/21645515.2014.1004030 25692455 PMC4514251

[20] Wilson L, Rubens-Augustson T, Murphy M, et al. Barriers to immunization among newcomers: A systematic review. *Vaccine* 2018; 36: 1055–1062. doi: 10.1016/j.vaccine.2018.01.025 29395515

[21] Perman S, Turner S, Ramsay AIG, et al. School-based vaccination programmes: a systematic review of the evidence on organisation and delivery in high income countries. *BMC Public Health* 2017; 17: 252. doi: 10.1186/s12889-017-4168-0 28288597 PMC5348876

[22] Selvey LA, Roux F, Burns S. Potential process improvements to increase coverage of human papillomavirus vaccine in schools–A focus on schools with low vaccine uptake. *Vaccine* 2020; 38: 2971–2977. doi: 10.1016/j.vaccine.2020.02.047 32115296

[23] Brewer NT, Fazekas KI. Predictors of HPV vaccine acceptability: A theory-informed, systematic review. *Prev Med (Baltim)* 2007; 45: 107–114. doi: 10.1016/j.ypmed.2007.05.013 17628649

[24] Jeudin P, Liveright E, del Carmen MG, et al. Race, ethnicity and income as factors for HPV vaccine acceptance and use. *Hum Vaccin Immunother* 2013; 9: 1413–1420. doi: 10.4161/hv.24422 23571170

[25] Remes O, Smith LM, Alvarado-Llano BE, et al. Individual- and regional-level determinants of human papillomavirus (HPV) vaccine refusal: the Ontario Grade 8 HPV vaccine cohort study. *BMC Public Health* 2014; 14: 1047. doi: 10.1186/1471-2458-14-1047 25297055 PMC4210569

[26] Smith LM, Brassard P, Kwong JC, et al. Factors associated with initiation and completion of the quadrivalent human papillomavirus vaccine series in an ontario cohort of grade 8 girls. *BMC Public Health* 2011; 11: 645. doi: 10.1186/1471-2458-11-645 21838921 PMC3224094

[27] Liu XC, Bell CA, Simmonds KA, et al. HPV Vaccine utilization, Alberta 2008/09–2013/14 School year. *BMC Infect Dis* 2016; 16: 15. doi: 10.1186/s12879-016-1340-6 26759056 PMC4711033

[28] Walker TY, Elam-Evans LD, Yankey D, et al. National, Regional, State, and Selected Local Area Vaccination Coverage Among Adolescents Aged 13–17 Years—United States, 2017. *MMWR Morb Mortal Wkly Rep* 2018; 67: 909–917. doi: 10.15585/mmwr.mm6733a1 30138305 PMC6107323

[29] Williams CL, Walker TY, Elam-Evans LD, et al. Factors associated with not receiving HPV vaccine among adolescents by metropolitan statistical area status, United States, National Immunization Survey–Teen, 2016–2017. *Hum Vaccin Immunother* 2020; 16: 562–572. doi: 10.1080/21645515.2019.1670036 31584312 PMC7227662

[30] Crosby RA, Casey BR, Vanderpool R, et al. Uptake of free HPV vaccination among young women: a comparison of rural versus urban rates. *J Rural Heal* 2011; 27: 380–384. doi: 10.1111/j.1748-0361.2010.00354.x 21967381 PMC4753792

[31] Sell H, Paudel YR, Voaklander D, et al. School immunization coverage during the COVID-19 pandemic: A retrospective cohort study. *medRxiv* 2022; 2022.05.04.22274665. 10.1016/j.vaccine.2023.01.011 PMC9826994 36642632

[32] Piché-Renaud P-P, Ji C, Farrar DS, et al. Impact of the COVID-19 pandemic on the provision of routine childhood immunizations in Ontario, Canada. *Vaccine* 2021; 39: 4373–4382. doi: 10.1016/j.vaccine.2021.05.094 34108076 PMC9756828

[33] Alberta Health Services. Human Papillomavirus 9-valent Vaccine (HPV 9), https://open.alberta.ca/dataset/58d31634-61d9-469d-b95f-f714719b923e/resource/aaec771b-7add-4d50-97e8-9a7b5f8c9ddf/download/aip-bp-hpv-9.pdf (2020, accessed 7 September 2021).

[34] Health Alberta. Interactive Health Data Application, http://www.ahw.gov.ab.ca/IHDA_Retrieval/selectSubCategory.do (accessed 7 September 2021).

[35] Ivankova N V, Creswell JW, Stick SL. Using Mixed-Methods Sequential Explanatory Design: From Theory to Practice. *Field methods* 2006; 18: 3–20.

[36] Levesque J-F, Harris MF, Russell G. Patient-centred access to health care: conceptualising access at the interface of health systems and populations. *Int J Equity Health* 2013; 12: 18. doi: 10.1186/1475-9276-12-18 23496984 PMC3610159

[37] Alberta Health Services. About AHS, https://www.albertahealthservices.ca/about/about.aspx (accessed 7 September 2021).

[38] Alberta Health Services. Immunization—School Services, https://www.albertahealthservices.ca/findhealth/service.aspx?id=4209.

[39] Sullivan KM, Pezzullo JC, Dean AG. Sample Size for a Proportion or Descriptive Study, https://www.openepi.com/SampleSize/SSPropor.htm (2013, accessed 22 September 2021).

[40] Bolarinwa OA. Principles and methods of validity and reliability testing of questionnaires used in social and health science researches. *Niger Postgrad Med J* 2015; 22: 195. doi: 10.4103/1117-1936.173959 26776330

[41] Helmkamp LJ, Szilagyi PG, Zimet G, et al. A validated modification of the vaccine hesitancy scale for childhood, influenza and HPV vaccines. *Vaccine* 2021; 39: 1831–1839. doi: 10.1016/j.vaccine.2021.02.039 33676784 PMC13325669

[42] Pleasant A, Maish C, O’Leary C, et al. A theory-based self-report measure of health literacy: The Calgary Charter on Health Literacy scale. *Methodol Innov* 2018; 11: 2059799118814394.

[43] World Health Organization. Vaccine Hesitancy Survey Questions Related to SAGE Vaccine Hesitancy Matrix, https://www.who.int/immunization/programmes_systems/Survey_Questions_Hesitancy.pdf?ua=1 (accessed 7 September 2021).

[44] Lemstra M, Neudorf C, Opondo J, et al. Disparity in childhood immunizations. *Paediatr Child Health (Oxford)* 2007; 12: 847–852. doi: 10.1093/pch/12.10.847 19043498 PMC2532573

[45] Larson HJ, Jarrett C, Schulz WS, et al. Measuring vaccine hesitancy: The development of a survey tool. *Vaccine* 2015; 33: 4165–4175. doi: 10.1016/j.vaccine.2015.04.037 25896384

[46] Fisher WA, Kohut T, Salisbury CMA, et al. Understanding Human Papillomavirus Vaccination Intentions: Comparative Utility of the Theory of Reasoned Action and the Theory of Planned Behavior in Vaccine Target Age Women and Men. *J Sex Med* 2013; 10: 2455–2464. doi: 10.1111/jsm.12211 23745833

[47] Stout ME, Christy SM, Winger JG, et al. Self-efficacy and HPV Vaccine Attitudes Mediate the Relationship Between Social Norms and Intentions to Receive the HPV Vaccine Among College Students. *J Community Health* 2020; 45: 1187–1195. doi: 10.1007/s10900-020-00837-5 32418009 PMC7606315

[48] Damnjanović K, Graeber J, Ilić S, et al. Parental Decision-Making on Childhood Vaccination. *Frontiers in Psychology* 2018; 9: 735. 29951010 10.3389/fpsyg.2018.00735 PMC6008886

[49] Gust DA, Kennedy A, Shui I, et al. Parent Attitudes Toward Immunizations and Healthcare Providers: The Role of Information. *Am J Prev Med* 2005; 29: 105–112. doi: 10.1016/j.amepre.2005.04.010 16005806

[50] Harris PA, Taylor R, Thielke R, et al. Research electronic data capture (REDCap)—a metadata-driven methodology and workflow process for providing translational research informatics support. *J Biomed Inform* 2009; 42: 377–381. doi: 10.1016/j.jbi.2008.08.010 18929686 PMC2700030

[51] Harris PA, Taylor R, Minor BL, et al. The REDCap consortium: Building an international community of software platform partners. *J Biomed Inform* 2019; 95: 103208. doi: 10.1016/j.jbi.2019.103208 31078660 PMC7254481

[52] Davies C, Stoney T, Hutton H, et al. School-based HPV vaccination positively impacts parents’ attitudes toward adolescent vaccination. *Vaccine* 2021; 39: 4190–4198. doi: 10.1016/j.vaccine.2021.05.051 34127299

[53] Bair RM, Mays RM, Sturm LA, et al. Acceptability of the Human Papillomavirus Vaccine among Latina Mothers. *J Pediatr Adolesc Gynecol* 2008; 21: 329–334. doi: 10.1016/j.jpag.2008.02.007 19064226

[54] King N, Brooks J, Tabari S. Template analysis in business and management research. In: *Qualitative methodologies in organization studies*. Springer, 2018, pp. 179–206.

[55] Statistics Canada. Focus on Geography Series, 2016 Census, https://www12.statcan.gc.ca/census-recensement/2016/as-sa/fogs-spg/Facts-pr-eng.cfm?Lang=Eng&GK=PR&GC=48&TOPIC=7 (2019).

[56] Alberta Health Services. Consent to Treatment/Procedure(s): Minors/Mature Minors, https://extranet.ahsnet.ca/teams/policydocuments/1/clp-consent-to-treatment-prr-01-03-procedure.pdf (2020, accessed 7 September 2021).

